# *Rhizobium radiobacter* peritonitis: the first case report from India and review

**DOI:** 10.1099/jmmcr.0.004051

**Published:** 2014-12-01

**Authors:** Richa Misra, Kashi Nath Prasad, Kamini Singh, Dharmendra Bhadauria, R. K Sharma

**Affiliations:** ^1^​ Department of Microbiology, Sanjay Gandhi Postgraduate Institute of Medical Sciences, Lucknow, India; ^2^​ Department of Nephrology, Sanjay Gandhi Postgraduate Institute of Medical Sciences, Lucknow, India

**Keywords:** abdominal pain, antibiotics, catheter removal, CAPD, cloudy effluent, fever, peritonitis, *Rhizobium radiobacter*

## Abstract

**Background:**

*Rhizobium radiobacter* is an opportunistic human pathogen in debilitated patients with foreign plastic intravascular devices and peritoneal dialysis (PD) catheters. We performed a Medline search of the English-language literature on *R. radiobacter* continuous ambulatory peritoneal dialysis (CAPD) peritonitis in end-stage renal disease (ESRD) and reviewed 13 cases.

**Case presentation:**

A 54-year-old male with ESRD secondary to chronic long-standing type II diabetes mellitus presented to the nephrology outpatient department with fever, abdominal pain and cloudy PD effluent. The patient was admitted to the hospital. PD fluid was sent for cell count, Gram stain and culture. The white blood cell (WBC) count in the PD fluid was 6400 mm^−3^ with 82 % neutrophils. Gram staining of the fluid showed plenty of Gram-negative bacilli. A presumptive diagnosis of CAPD peritonitis was made and empiric intraperitoneal cefazolin and tobramycin were started. The PD fluid culture grew non-fermenting, Gram-negative bacilli identified as *R. radiobacter.* Empiric antibiotic therapy was continued and the patient’s abdominal pain subsided. The peritoneal fluid counts decreased to 50 WBCs mm^−3^ on day 4. However, his abdominal pain recurred on day 8 and his PD fluid count increased to 300 cells mm^−3^. The catheter was therefore surgically removed.

**Conclusion:**

Although *R. radiobacter* is considered a contaminant, it can cause relapsing symptomatic peritonitis peritoneal catheter removal.

## Background

*Staphylococcus epidermidis* and *Staphylococcus aureus* are the most common pathogens causing peritonitis in peritoneal dialysis (PD) patients (Kam–Tao Li *et al*., 2010). They are followed by Gram-negative organisms and fungi. However, recently there have been increasing reports of rare and unusual organisms causing peritonitis in patients maintained on PD (Imran *et al*., 2012; Kahveci *et al*., 2011).Strains of *Rhizobium* spp. when recovered from clinical specimens are considered contaminants or organisms of low pathogenic potential (Edmond *et al*., 1993). They are aerobic, motile, oxidase-positive, non-spore-forming, Gram-negative bacilli. The genus *Agrobacterium* belongs to the family *Rhizobiaceae* in subphylum alpha of the proteobacteria. 16S rRNA gene-based phylogeny grouped the genus *Agrobacterium* and some fast-growing, nitrogen-fixing bacteria of the genus *Rhizobium* in the same clade. Usually, clinical strains of the genus *Agrobacterium* are affiliated with the species *Agrobacterium radiobacter*, whereas *Agrobacterium tumefaciens* includes environmental phytopathogenic and non-phytopathogenic strains (Aujoulat *et al*., 2011).Although their recovery from human clinical specimens was noted in 1967, the first clinical case infection reported was prosthetic valve endocarditis in 1980 (Kersters & de Ley, 1984; Lautrop, 1967; Plotkin, 1980). *R. radiobacter* has only recently been recognized as an opportunistic human pathogen in immunocompromised and debilitated patients with foreign plastic intravascular and PD catheters (Rodby & Glick 1991). Bacteraemia, peritonitis and urinary tract infections are the most common clinical syndromes reported with this organism (Hulse *et al.*, 1993). To the best of our knowledge, this case is the first report from India of continuous ambulatory peritoneal dialysis (CAPD) peritonitis caused by *Rhizobium radiobacter*. Although our isolate was sensitive to most of the antimicrobial agents used and the patient initially improved with appropriate antibiotics, the catheter had to be surgically removed. In addition, we reviewed 13 cases of CAPD peritonitis caused by *R. radiobacter* since 1900 and divided the clinical course into those who continued on PD after appropriate antibiotics and those who relapsed soon afterwards, necessitating the eventual removal of the catheter and outpatient haemodialysis.

## Case report

A 54-year-old male with end-stage renal disease (ESRD) secondary to chronic, long-standing type II diabetes mellitus presented in January 2011 with decreased urine output and swelling over the whole body for 3 months. He was trained for CAPD in a stable state of health. The patient was compliant with medical care and had been free from peritonitis to date. However, he presented to the nephrology outpatient department in January 2013 with complaints of fever, abdominal pain and cloudy PD effluent. As the abdominal pain worsened, the patient was admitted to the hospital. The patient denied any contact with soil or plant material. His past medical history was uneventful except for diabetes mellitus for the past 11 years. On physical examination, his blood pressure was 140/80 mm Hg^−1^, temperature 100.8 ° F, pulse 72 min^−1^ and respiration rate 18 min^−1^. His head, eyes, ears, nose and throat examination was unremarkable. There was no clubbing, cyanosis, lymphadenopathy or icterus. His lungs were clear and no cardiac murmur was heard. His abdomen was tender in all quadrants with rebound tenderness. Bowel sounds were present and active. Pertinent laboratory values were Hgm (Hemoglobin) 7.2 gm dl^−1^, white blood cells (WBCs) 24000 mm^−3^, platelets 2.28×10^5^ µl^−1^, serum creatinine 9.3 mg dl^−1^, serum blood urea nitrogen 45 mg dl^−1^, serum uric acid 4.9 mg dl^−1^, serum sodium 131 mmol l^−1^, serum potassium 3.5 mmol l^−1^, serum albumin 3.3 g dl^−1^, serum bilirubin (total) 0.45 mg dl^−1^, serum aspartate transaminase 19 U l^−1^, serum alanine aminotransferase 13 U L^−1^, serum alkaline phosphatase 105 U l^−1^, serum amylase 29 U L^−1^, serum calcium 8.9 mg dl^−1^, serum phosphorus 5 mg dl^−1^ and serum lactate dehydrogenase 514 U l^−1^. PD fluid was sent for cell count, Gram stain and culture. The WBC count in PD fluid was 6400 mm^−3^ with 82% neutrophils. Gram staining of the fluid showed plenty of Gram-negative bacilli. A presumptive diagnosis of CAPD peritonitis was made and empiric intraperitoneal cefazolin and tobramycin were started. PD fluid culture yielded catalase-positive, oxidase-positive, non-spore-forming and non-fermenting Gram-negative bacilli. The isolate was positive for urea hydrolysis and nitrate reduction. Negative reactions were found for the production of H_2_S, indole, lysine decarboxylase and ornithine decarboxylase for the hydrolysis of gelatin. The identity of the isolate was confirmed as *R. radiobacter* sensitive to trimethoprim/sulfametoxazole, ceftazidime, ceftriaxone, ampicillin-sulbactam, cefoperazone-sulbactam, piperacillin-tazobactam, amikacin, gentamicin, tobramycin, ciprofloxacin, imipinem and meropenem. It was resistant only to aztreonam. Empiric antibiotic therapy was continued and the patient’s abdominal pain subsided and the peritoneal fluid counts decreased to 50 WBCs mm^−3^ on day 4. However, on the eighth hospital day, the patient again complained of abdominal pain and the PD fluid count increased to 300 WBCs mm^−3^. The catheter was therefore surgically removed and the tip sent for culture. The tip yielded growth of *R. radiobacter*.

## Methods

We performed a Medline search of the English-language literature on *R. radiobacter* CAPD peritonitis in ESRD and reviewed reference lists for additional cases published since the year 1900. All cases with the following criteria were included in our review: (i) the patient had clinical evidence of peritonitis (fever, abdominal pain and cloudy effluent); (ii) bacterial proof of culture of PD fluid yielded *R. radiobacter*. Documentation of the relevant clinical information included underlying disease causing (ESRD), associated medical condition such as use of an indwelling medical catheter, antibiotic regimens received, susceptibility pattern of the isolate and outcome. A history of contact with plant or soil material was also noted.

The isolate recovered from our patient was identified as *R. radiobacter* on the basis of colony morphology, motility, Gram-staining characteristics, oxidase reaction, growth on triple-sugar agar and biochemical profile. It was confirmed as *R. radiobacter* by the biochemical profile obtained using the Phoenix System, BD Diagnostics (Becton, Dickinson and Company). The isolate was submitted for sequencing in the National Centre for Biotechnology Information, NCBI and the accession number is KM 583853.

## Results

The clinical characteristics of the 13 patients are provided in Table 1. Nine cases were male and four female. The mean age of the patients was 45.2 years (range, 11–66 years). Risk based on age, gender and duration of PD therapy was not observed. All patients presented with fever, abdominal pain and cloudy peritoneal effluent. All cases appeared to have been community acquired, although a history of contact with plant or soil material was elicited in only two cases. All 13 patients from whom *R. radiobacter* was recovered had significant underlying illness (Table 1) causing ESRD. Associated medical conditions such as diabetes mellitus were present in one case, whilst two patients each were hypertensive and smokers. One case occurred in an 11-year-old girl affected by Down’s syndrome, while IgA nephropathy, polycystic kidney disease, renal amyloidosis and nephroangiosclerosis were present in one case each. All patients were treated with appropriate antibiotics after PD fluid cultures were reported to be positive for *R. radiobacter*. *In vitro* susceptibility to antibiotics in all the reported cases was variable. Antibiotic susceptibility was not available for one isolate due to scanty growth (Rodby & Glick, 1991). Five isolates were resistant to tobramycin and necessitated a change in antibiotic therapy. The initial response to the appropriate antibiotic administered after susceptibility testing was favourable in all the cases. However, eventual removal of the intraperitoneal catheter was regarded as the definitive treatment in eight patients, whilst five patients were treated successfully with antibiotics. One patient underwent a transplant after recovery.

**Table 1.** Clinical and demographic characteristics of 13 patients with *R. radiobacter* peritonitis

Reference	Age/gender	Underlying condition	Contact with plant/soil	Sensitivity	Empiric treatment	Management	Outcome
Rodby & Glick (1991) (two cases)	66 years/F	HTN	No	na (due to scanty growth)	Vancomycin and amikacin; later co-trimoxazole	Removal of catheter and HD	Alive and on outpatient HD
	71 years/M	DM, HTN	No	Resistant: amikacin, gentamicin, tobramycin, ceftazidime, cefuroxime, cephalothin; sensitive: cefoxitin, ceftriaxone, ciprofloxacin, impinem, piperacillin, ticarcillin-clavulanic acid, tetracycline and cotrimoxazole	Vancomycin and tobramycin; later cefoxitin	Removal of catheter and HD	Alive and on outpatient HD
Hulse *et al*. (1993)(one case)	20 years/M	Membrano-proliferativeglomerulo-nephritis	na (community acquired)	Sensitive: penicillins, cephalosporins, trimethoprim-sulfametoxazole, tetracycline, gentamicin and amikacin; Resistant: tobramycin	ip gentamicin and trimethoprim-sulfametoxazole and iv gentamicin and ticarcillin	Treated successfully with antibiotics	Alive and continued on CAPD
Alnor *et al*. (1994) (three cases)	56 years/F	Renal hyperplasia	na	Sensitive: ciprofloxacin, imipenem and tetracycline; resistant: ceftazidime, piperacillin and aztreonam		Removal of catheter	Alive
	31 years/F	Renal amyloidosis	na	As above		Removal of catheter	Alive
	61 years/F	Glomerulonephritis	na	As above		Treated successfully with antibiotics	Alive
Melgosa *et al*. (1997) (one case)	11 years/F	Down’s syndrome(reflux nephropathy)	No	Sensitive: imipenem; resistant: tobramycin	Vancomycin and tobramycin (i.p.); later imipinem	Removal of catheterand HD	Alive and on HD
Lui & Lo, (2005)(one case)	43 years/M	PCKD	No	Sensitive: netilmycin; resistant: cefazolin	Cefuroxime and netilmycin	Removal of catheter and HD	Alive and on HD
Minguela *et al*., (2006) (one case)	63 years/M	Nephroangiosclerosis	No	Resistant: tobramycin, ceftazidime; sensitive: amoxicillin-clavulanic acid, cefotaxime, imipinem and ciprofloxacin	Vancomycin and ceftazidime; later amoxycillin-clavulanic acid and gentamicin, finally cefotaxime and ciprofloxacin	Continued on PD for 6 months	Transplanted and alive
Levitski–Heikkila & Ullian, 2005 (1 case)*	41 years/M	Smoking	Yes	Sensitive: all β-lactams, cephalosporins, ciprofloxacin and gentamicin	Cefazolin and gentamicin	Removal of catheter and HD	Death after 1 month
Rothe & Rothenpieler, 2007 (one case)	41 years/M	ESRD	No	Resistant: tobramycin and cotrimoxazole; sensitive: imipinem and cefepime	Cefepime and ciprofloxacin	Removal of catheter	Alive
Han & Han, (2007) (one case)	42 years/M			Sensitive to all antibiotics	Ceftazidime and ciprofloxacin	Treated successfully with antibiotics	Alive and no relapse till 1 year follow-up
Tsai (2013) (one case)	42 years/M	IgA nephropathy	Yes	Resistant: gentamicin and cotrimoxazole	Cefazolin and ceftazidime	Treated successfully with antibiotics	Alive and no relapse till 2 year follow-up

M, male; F, female; NA, not available; DM, diabetes mellitus; HD, haemodialysis; HTN, hypertension; i.v., intravenous; i.p. intraperitoneal; PCKD, polycystic kidney disease.

* Co-infection with R. *radiobacter* and *Pseudomonas oryzihabitans.*

## Discussion

*R. radiobacter* is an aerobic, motile, Gram-negative bacterium found primarily in the soil (Edmond *et al*., 1993). It was formerly classified in the genus *Agrobacterium*, and differentiation of the phenotypically indistinguishable species *A. tumefaciens* and *A. radiobacter* was based on the presence of a plant tumour-inducing plasmid, present in *A. tumefaciens* and absent in *A. radiobacter* (Aujoulat *et al*., 2011). Genetic studies later revealed that the two species were the same, and a proposal was made to reject the name *A. tumefaciens* and to designate *A. radiobacter* as the type species for the genus *Agrobacterium*. Young *et al*. (2003) proposed an amended description of the genus *Rhizobium* to include all species of *Agrobacterium*. *R. radiobacter* has only recently been recognized as an opportunistic pathogen and when isolated from a clinical specimen is often considered a contaminant. Until the 1980s, investigators found little evidence to support a pathogenic role for these organisms in patients from whom they were recovered (Vanderleen *et al*., 1985). Our literature search on CAPD peritonitis revealed 13 case reports in which *R. radiobacter* was implicated as the causative pathogen. To the best of our knowledge, this is the first case report from India of CAPD peritonitis caused by *R. radiobacter.* As patients with ESRD have an altered immune status and the peritoneal cavity filled with dialysate is an excellent culture medium for micro-organisms, even rare isolates can cause peritonitis in chronic dialysis. In our patient, the exact source of *R. radiobacter* causing peritonitis could not be ascertained and, although a history of contact with soil was not elicited, the infection seemed to be community acquired. Although the number of reported cases is limited and evidence in literature is sparse, a certain pattern of PD peritonitis due to *R. radiobacter* may be postulated. The clinical presentation of all the reported cases was similar to those observed in peritonitis caused by more-common organisms. All patients presented with fever, abdominal pain, tenderness and cloudy effluent, and all initially responded well to antibiotics. However, the clinical course could be divided into those who continued on peritoneal dialysis after appropriate antibiotics and those who relapsed soon afterwards, necessitating the eventual removal of the catheter and outpatient haemodialysis. Lui & Lo (2005) reported the case of a 43-year-old Chinese ESRD patient where they describe a relapsing peritonitis and recommend removing the catheter as soon as possible. A similar outcome was reported by Rothe & Rothenpieler (2007) in a multidrug-resistant strain of *R. radiobacter* causing peritonitis in a 41-year-old Caucasian male with ESRD. However, preservation of the catheter was reported by Tsai (2013) in a patient with IgA nephropathy maintained on intraperitoneal ceftazidime for 3 weeks.

An insight into the antimicrobial susceptibility test results revealed that, although all the reported cases were community acquired, each isolate was resistant to at least one antibiotic agent. All the reviewed strains were, however, susceptible to ciprofloxacin and imipenem. Our isolate was resistant to aztreonam. Previous findings suggest that clinical isolates of *Rhizobium* spp. that are resistant to aztreonam are common because monobactams can be produced by some soil strains of *Rhizobium*. Martinez *et al.* (1989) showed one clinical isolate to contain an inducible cephalosporinase, an aminoglycoside acetyl transferase and a chloramphenicol acetyl transferase. The PD-related infection recommendations (Kam–Tao Li *et al*., 2010) state that empiric antibiotics must cover both Gram-positive and Gram-negative organisms. The Committee recommends centre-specific selection of empiric therapy, dependent on the local history of sensitivities of organisms causing peritonitis. Intraperitoneal administration of antibiotics is superior to intravenous dosing for treating peritonitis. Therapy is initiated prior to knowledge of the causative organism in light of local microbiological data and should be initiated as soon as possible after appropriate microbiological specimens have been obtained. A first-generation cephalosporin, such as cefazolin or cephalothin, with a second drug for broader Gram-negative coverage (including coverage for *Pseudomonas*) should prove suitable. Many institutions have a high rate of meticillin-resistant organisms and thus should use vancomycin. Vancomycin resistance is, however, obligate in *R. radiobacter* and, being a biofilm inhabitant, it is potentially difficult to eradicate. The fact that eight patients did not recover until their catheters had been removed may indicate that the devices were colonized. Contamination of catheters was the most evident cause of infection in the reviewed cases. *Radiobacter* spp. are ubiquitous in soil but are not part of the indigenous human flora. Patients with permanent catheters may become infected when they handle plants at home or outside. All other reported infections with *R. radiobacter* apart from PD peritonitis were also associated with artificial materials such as central venous catheters (Paphitou & Rolston, 2003), prosthetic heart valves (Plotkin, 1980) and lens implants (Namdari *et al*., 2003). All these devices were eventually removed.

To conclude, *R. radiobacter* is an opportunistic community-acquired pathogen in CAPD patients and there is no consensus regarding catheter removal in this kind of infection. Treatment should be guided by the susceptibility profile of the individual isolate. In case of relapse, it is advisable to remove the catheter.

## Abbreviations

CAPD: continuous ambulatory peritoneal dialysis
ESRD: end-stage renal disease
PD: peritoneal dialysis.
